# Deactivation of Glutaminolysis Sensitizes *PIK3CA*-Mutated Colorectal Cancer Cells to Aspirin-Induced Growth Inhibition

**DOI:** 10.3390/cancers12051097

**Published:** 2020-04-30

**Authors:** Shogen Boku, Motoki Watanabe, Mamiko Sukeno, Takeshi Yaoi, Kiichi Hirota, Mahiro Iizuka-Ohashi, Kyoko Itoh, Toshiyuki Sakai

**Affiliations:** 1 Department of Molecular-Targeting Prevention, Kyoto Prefectural University of Medicine, Kyoto 602-8566, Japan; bokusho@koto.kpu-m.ac.jp (S.B.); iom0220@koto.kpu-m.ac.jp (M.I.-O.); 2 Drug Discovery Center, Kyoto Prefectural University of Medicine, Kyoto 602-8566, Japan; sukeno@koto.kpu-m.ac.jp (M.S.); tsakai@koto.kpu-m.ac.jp (T.S.); 3 Department of Pathology and Applied Neurobiology, Graduate School of Medical Science, Kyoto Prefectural University of Medicine, Kyoto 602-8566, Japan; tyaoi@koto.kpu-m.ac.jp (T.Y.); kxi14@koto.kpu-m.ac.jp (K.I.); 4 Department of Human Stress Response Science, Institute of Biomedical Science, Kansai Medical University, Hirakata 573-1010, Japan; hif1@mac.com; 5 Department of Endocrine and Breast Surgery, Kyoto Prefectural University of Medicine, Kyoto 602-8566, Japan

**Keywords:** aspirin, *PIK3CA*, glutaminolysis, ATF4, colorectal cancer

## Abstract

Aspirin is one of the most promising over-the-counter drugs to repurpose for cancer treatment. In particular, aspirin has been reported to be effective against *PIK3CA*-mutated colorectal cancer (CRC); however, little information is available on how the *PIK3CA* gene status affects its efficacy. We found that the growth inhibitory effects of aspirin were impaired upon glutamine deprivation in *PIK3CA*-mutated CRC cells. Notably, glutamine dependency of aspirin-mediated growth inhibition was observed in *PIK3CA*-mutated cells but not *PIK3CA* wild type cells. Mechanistically, aspirin induced G1 arrest in *PIK3CA*-mutated CRC cells and inhibited the mTOR pathway, inducing the same phenotypes as glutamine deprivation. Moreover, our study including bioinformatic approaches revealed that aspirin increased the expression levels of glutaminolysis-related genes with upregulation of activating transcription factor 4 (ATF4) in *PIK3CA*-mutated CRC cells. Lastly, the agents targeting glutaminolysis demonstrated significant combined effects with aspirin on *PIK3CA*-mutated CRC cells. Thus, these findings not only suggest the correlation among aspirin efficacy, *PIK3CA* mutation and glutamine metabolism, but also the rational combinatorial treatments of aspirin with glutaminolysis-targeting agents against *PIK3CA*-mutated CRC.

## 1. Introduction

Non-steroidal anti-inflammatory drugs (NSAIDs) have held great promise as a repurposed drug for the prevention and treatment of cancer, especially colorectal cancer (CRC) [1,2]. Notably, randomized trials of aspirin for the prevention of cardiovascular and cerebrovascular events suggested that regular aspirin use could reduce the incidence and mortality of CRC [3,4]. As for the treatment for CRC, a recent study suggested that regular use of aspirin after CRC diagnosis can prolong the survival of patients with *PIK3CA* active mutations [5], and this was confirmed by other large cohort studies [6,7]. Thus, the *PIK3CA* status may be a predictor to stratify CRC patients who will benefit from adjuvant therapy using aspirin.

*PIK3CA* mutations are observed among 10%–20% of CRC cases [8] and influence its malignant features. For example, *PIK3CA* mutations in two hot spots in both exons 9 and 20 may be associated with a poorer prognosis [9,10]. As another example, *PIK3CA* mutations negatively impact the response of CRC to first-line chemotherapies, including FOLFOX, XELOX and FOLFIRI [11] and anti-EGFR-targeted therapies [12]. Furthermore, *PIK3CA* mutations induce metabolic vulnerabilities in CRC cells. Whereas the growth of *PIK3CA*-mutated cancer including CRC is dependent on glucose metabolism [13], recent reports demonstrated that *PIK3CA* mutations in CRC confer the dependency on glutamine metabolism [14], which may be targetable to suppress the growth of *PIK3CA*-mutated CRC [15].

These observations mentioned above imply relationships among the status of the *PIK3CA* gene, metabolic dependency and the sensitivity to aspirin in CRC cells; however, the impact of aspirin on cancer metabolism and how it affects the sensitivity to aspirin in *PIK3CA*-mutated CRC is not well characterized. In the present study, we found that aspirin requires glutamine to inhibit cell growth in *PIK3CA*-mutated CRC cells. Furthermore, we demonstrated that aspirin promotes glutaminolysis partially due to activating transcription factor 4 (ATF4)-driven transcriptional program. Lastly, based on these findings, we propose the rationalized combinatorial strategy of aspirin with glutaminolysis-targeting agents against *PIK3CA*-mutated CRC.

## 2. Results

### 2.1. Aspirin-Mediated Growth Inhibition is Dependent on Glutamine in PIK3CA-Mutated Cells

**Figure 1:**
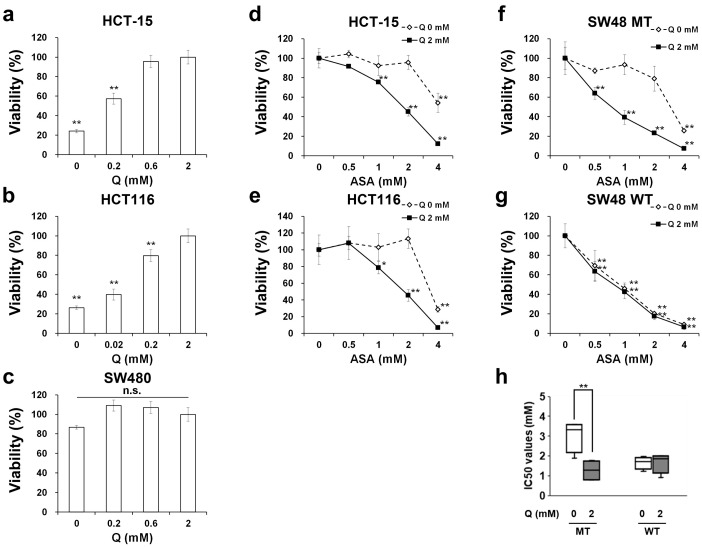
Aspirin-mediated growth inhibition depends on glutamine in *PIK3CA*-mutated cells. (**a–c)** Glutamine dependency of cell growth. HCT-15 (*PIK3CA* MT) (**a**), HCT116 (*PIK3CA* MT) (**b**) and SW480 (*PIK3CA* WT) (**c**) cells were incubated with glutamine (Q) at the indicated concentrations for 72 hr. Cell viability was measured by the Cell Counting Kit-8 assay. The data obtained with 2 mM Q were taken as 100%. Columns, means (*n* = 3); bars, s.d. ** *p* < 0.01, significantly different from the 2 mM Q-treated control. (**d–g**) Glutamine dependency of the inhibitory effects of aspirin (ASA) on growth in *PIK3CA* wild type and mutated cells. HCT-15 (**d**), HCT116 (**e**), SW48 MT (*PIK3CA* MT) (**f**) and SW48 WT (*PIK3CA* WT) (**g**) cells were treated with ASA at the indicated concentrations for 72 hr with or without 2 mM Q. The data obtained with dimethyl sulfoxide (DMSO)-treated controls were taken as 100%. Columns, means (*n* = 3); bars, s.d. * *p* < 0.05, ** *p* < 0.01, significantly different from the DMSO-treated control. (**h**) The comparison of IC50 values of ASA in *PIK3CA* MT and WT cells incubated with or without glutamine. The IC50 values of ASA in SW48 *PIK3CA*-mutated and wild type cells incubated with or without 2 mM Q were calculated. The grouped IC50 values from three independent experiments are shown as box plots. Statistical analyses were performed using one-way ANOVA with Tukey’s post-hoc test (**a**–**g**) and two-tailed *t*-test (**h**).

It was previously reported that glutamine deprivation significantly reduces the proliferation of *PIK3CA* mutant, but not the wild type, cancer cells [14]. Consistent with this, human CRC HCT-15 and HCT116 cells harboring *PIK3CA* mutations accelerated cell growth in a glutamine dose-dependent manner (Figure 1a,b), whereas *PIK3CA* wild type human CRC SW480 cells were not affected by glutamine supplementation (Figure 1c). We then examined whether the sensitivity to aspirin is affected by glutamine deprivation and the gene status of *PIK3CA*. Glutamine deprivation partially impaired the growth inhibitory effects of aspirin in HCT-15 and HCT116 cells (Figure 1d,e and Figure S1a,b). To confirm these findings, we used isogenic human CRC parental SW48 cells and SW48 cell clones with the knock-in *PIK3CA* mutation. Glutamine dependency of aspirin-mediated growth inhibition was observed in *PIK3CA*-mutated SW48 cells but not wild type cells (Figure 1f,g and Figure S1c,d). Indeed, although glutamine deprivation did not affect the IC50 values of aspirin in *PIK3CA* wild type cells, the median IC50 value in *PIK3CA*-mutated cells was significantly higher under glutamine-deprived conditions than in normal medium culture conditions (Figure 1h). This suggests that aspirin requires glutamine to sufficiently suppress cell growth in *PIK3CA*-mutated cells.

### 2.2. Glutamine Depletion Mirrors the Effects of Aspirin on the Cell Cycle and the mTOR Pathway in PIK3CA-Mutated Cells

**Figure 2:**
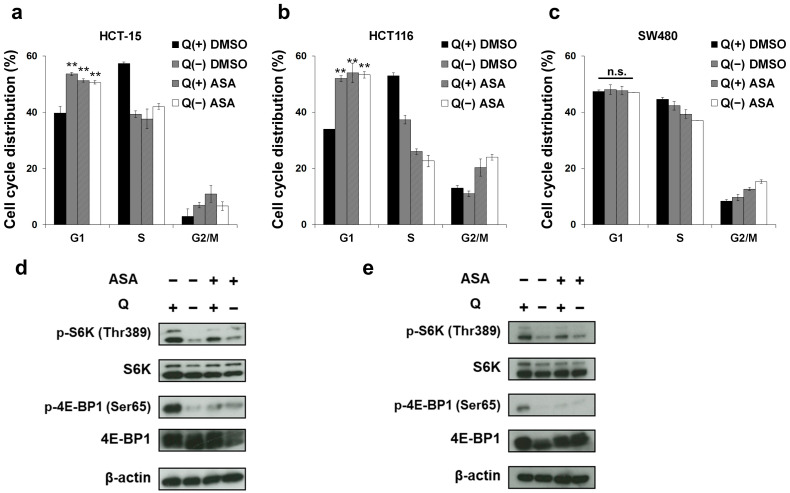
Aspirin and glutamine depletion induce G1 arrest and inhibit the mTOR pathway. (**a–c**) Cell cycle analysis of HCT-15, HCT116 and SW480 cells treated with aspirin (ASA) with or without glutamine (Q). HCT-15 (**a**), HCT116 (**b**) and SW480 (**c**) cells were treated with dimethyl sulfoxide (DMSO) or 2 mM ASA with or without 2 mM Q. After being incubated for 48 hr, the DNA content of the cells was measured by flow cytometry. The percentages of cells in the G1, S and G2/M phases of the cell cycle are shown. Columns, means (*n* = 3); bars, s.d. ** *p* < 0.01, significantly different from the DMSO-treated control with Q. (**d**,**e**) Effects on the mTOR pathway in *PIK3CA*-mutated cells treated with ASA with or without glutamine. Phosphorylated S6K at threonine 389, total S6K, phosphorylated 4E-BP1 at serine 65 and total 4E-BP1 were analyzed by Western blotting in HCT-15 (**d**) and HCT116 (**e**) cells treated with DMSO or 2 mM ASA with or without 2 mM Q for 48 hr. β-Actin was used as a loading control. Statistical analyses were performed using two-tailed *t*-test (**a–c**).

We next performed cell cycle analysis to investigate the mechanism of glutamine dependency of aspirin sensitivity in *PIK3CA*-mutated cells. First, in HCT-15 cells, glutamine deprivation induced G1 arrest (Figure 2a). Similar to glutamine deprivation, aspirin treatment also induced G1 arrest under normal culture conditions (Figure 2a). Of note, aspirin treatment in the glutamine-depleted medium did not cause additional accumulation of cells in the G1 phase (Figure 2a), suggesting that aspirin targets the same pathway as glutamine deprivation. The same trend was observed with HCT116 (Figure 2b), whereas the cell cycle distribution of SW480 cells was affected by neither aspirin nor glutamine deprivation (Figure 2c). As we did not observe the induction of cell death in these experimental settings (Figure S2a–c), it was considered that the induction of G1 arrest by aspirin contributed to the efficacy of aspirin and was specific to *PIK3CA* mutation. To further explore the common molecular mechanisms of aspirin and glutamine deprivation, we next performed Western blotting. As glutamine stimulates mTORC1 activation [16], we focused on the mTOR pathway. As expected, glutamine deprivation markedly inhibited the phosphorylation of the two major substrates of mTORC1, ribosomal protein S6 kinase (S6K) and eukaryotic translation initiation factor 4E-binding protein 1 (4E-BP1), in both HCT-15 (Figure 2d and Figure S6a) and HCT116 (Figure 2e and Figure S6b) cells. As with glutamine deprivation, aspirin also inhibited the phosphorylation of S6K and 4E-BP1 in both cell lines (Figure 2d,e). Taken together, these results demonstrate that aspirin and glutamine deprivation similarly target the mTOR pathway, whose inactivation may be required for the antitumor efficacy of aspirin in *PIK3CA*-mutated cells.

### 2.3. Aspirin Broadly Upregulates Glutaminolysis-Related Genes

**Figure 3:**
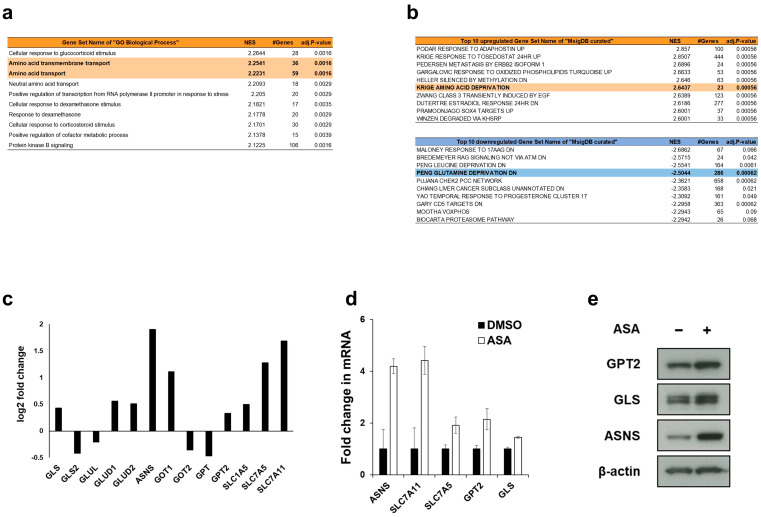
Aspirin upregulates glutaminolysis-related genes in *PIK3CA*-mutated cells. (**a**,**b**) Gene sets of ”GO biological process” (**a**) and “Molecular Signatures Database (MSigDB) Curated Gene Sets”(**b**) enriched in RNA-seq analysis of HCT-15 cells treated with 2 mM aspirin (ASA) for 24 hr. Gene sets with FDR *q*-value < 0.2 were considered as enriched. Top 10 normalized enrichment score (NES) were shown. (**c**) Global expression of glutaminolysis-related genes of publicly available microarray data in *PIK3CA*-mutated cells treated with ASA [20]. Logarithmic fold change of gene expression related to glutaminolysis in DLD-1 cells treated with 3 mM ASA compared with dimethyl sulfoxide (DMSO)-treated cells. (**d**) Confirmation of the upregulation of glutaminolysis-related genes in *PIK3CA*-mutated cells treated with ASA. The mRNA expression of glutaminolysis-related genes was measured by real-time RT–PCR in HCT-15 cells treated with DMSO or 2 mM ASA for 24 or 48 hr. Each mRNA level was normalized to that of β2MG, and the data obtained with DMSO-treated controls were taken as 1.0. Columns, means (*n* = 3); bars, s.d. (**e**) Confirmation of the upregulation of glutaminolysis-related proteins in *PIK3CA*-mutated cells treated with ASA. Glutaminolysis-related proteins were analyzed by Western blotting in HCT-15 cells treated with ASA for 48 hr. β-Actin was used as a loading control.

As the antitumor phenotype and molecular behavior of aspirin resemble those of glutamine deprivation, we hypothesized that aspirin can perturb glutamine metabolism. We then bioinformatically explored comprehensive gene expressions induced by aspirin. First, we performed RNA sequencing analysis of HCT-15 cells treated with DMSO or aspirin, and gene set enrichment analysis (GSEA) [17]. GSEA using the “GO Biological Process” datasets identified the pathways related to amino acid transport to be enriched in aspirin-treated cells (Figure 3a, Figure S3a,b). More interestingly, another “Molecular Signatures Database (MSigDB) Curated Gene Sets” showed that gene sets related to glutamine depletion were enriched in aspirin-treated cells (Figure 3b, Figure S4a,b). Second, we analyzed an available microarray dataset that evaluated the differential gene expressions induced by aspirin treatment in human CRC *PIK3CA*-mutated DLD-1 cells (as a genetically identical cell line to HCT-15 [18]) and *PIK3CA* wild type SW620 cells (as a cell line carrying identical mutation profiles to SW480 [19]) [20]. We noticed that the gene set of glutamine family amino acid catabolic process (GO:0009065) was enriched in gene ontology analysis in DLD-1 cells, while not in SW620 cells (Figure S5a,b). In this microarray experiment, glutaminolysis-related genes (i.e., *GLS*, *GLUD1*, *GLUD2*, *ASNS*, *GOT1*, *GPT2*, *SLC1A5*, *SLC7A5* and *SLC7A11*) were increased following aspirin treatment in DLD-1 cells (Figure 3c). We then successfully confirmed the increase in mRNA expressions of *ASNS*, *SLC7A11*, *SLC7A5*, *GPT2* and *GLS* genes induced by aspirin treatment (Figure 3d). Furthermore, aspirin induced the upregulation of GPT2, GLS and ASNS at the protein level (Figure 3e and Figure S6c). Taken together, aspirin may transcriptionally upregulate glutaminolysis-related genes in *PIK3CA*-mutated cells, mirroring the mode of actions of glutamine deprivation.

### 2.4. Aspirin Induces ATF4 Gene Expression via the ER Stress-Independent Mechanism

**Figure 4:**
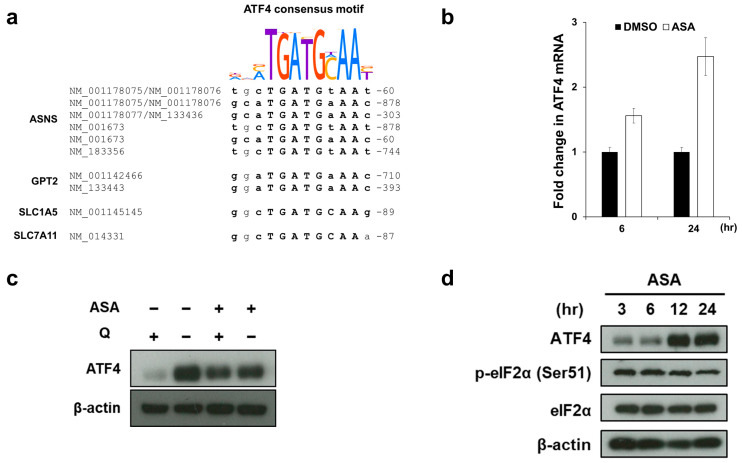
Aspirin induces ATF4 expression in *PIK3CA*-mutated cells. (**a**) Analysis of motif enrichment in the promoter sequences of the glutaminolysis-related genes. An ATF4 consensus motif was found in the promoters of *ASNS*, *GPT2*, *SLC1A5* and *SLC7A11*. RefSeq accession numbers of each transcript are shown. Matched consensus sequences are noted in capitals and highly conserved sequences are noted in bold. (**b**) Analysis of ATF4 mRNA induction in *PIK3CA*-mutated cells treated with aspirin (ASA). The expression of ATF4 mRNA was measured by real-time RT–PCR in HCT-15 cells treated with 2 mM ASA for 6 hr and 24 hr. ATF4 mRNA was normalized to β2MG mRNA, and the data obtained with dimethyl sulfoxide (DMSO)-treated controls were taken as 1.0. Columns, means (*n* = 3); bars, s.d. (**c**) ATF4 protein expressions in *PIK3CA*-mutated cells treated with ASA with or without glutamine. ATF4 expression was analyzed by Western blotting in HCT-15 treated with DMSO or 2 mM ASA for 48 hr with or without 2 mM glutamine (Q). β-Actin was used as a loading control. (**d**) Analysis of the relationship between ATF4 protein expression and ER stress. ATF4, phosphorylated eukaryotic initiation factor 2α (eIF2α) at serine 51 and total eIF2α were analyzed by Western blotting in HCT-15 cells treated with 2 mM ASA for the indicated times. β-Actin was used as a loading control.

We next investigated how aspirin upregulates the mRNA expression of glutaminolysis-related genes. As activating transcription factor 4 (ATF4) is reported to be upregulated by deprivation of glutamine [21,22], we speculated that ATF4 binds to promoters of the 13 glutaminolysis-related genes, which were analyzed by a microarray (Figure 3c), to upregulate their expressions. To test this possibility, we performed motif enrichment analysis of the promoter sequences and noted enrichment (*p*-value = 6.17 × 10^−3^) of the binding sites of ATF4 in four genes (*ASNS*, *GPT2*, *SLC1A5* and *SLC7A11*; Figure 4a). These binding site sequences were highly matched to the ATF4 consensus motif deduced from a large ChIP-seq dataset and well conserved (Figure 4a), suggesting that ATF4 directly controls their expression at the transcriptional level. We next investigated whether aspirin affects the expression level of ATF4. As shown in Figure 4b, 2 mM aspirin treatment time-dependently increased ATF4 expression at the mRNA level in HCT-15 cells. Of note, ATF4 upregulation induced by aspirin was to a similar degree as that by glutamine deprivation (Figure 4c and Figure S6d). Furthermore, to investigate the mechanism(s) of aspirin-induced ATF4 upregulation, we evaluated endoplasmic reticulum (ER) stress, which is known to be a major trigger of ATF4 induction [23]. However, no fluctuation in the phosphorylation of eIF2α (p-eIF2α) as an ER stress marker was observed (Figure 4d and Figure S6e). This suggests that aspirin induces ATF4 through ER stress-independent mechanism(s) to promote glutaminolysis.

### 2.5. Glutaminolysis-Targeting Agents Increase the Efficacy of Aspirin in PIK3CA-Mutated Cells

**Figure 5:**
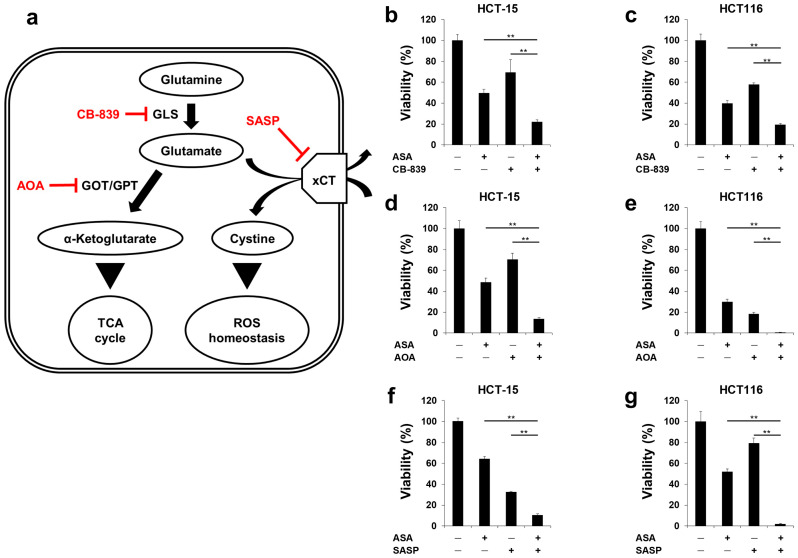
Glutaminolysis-targeting agents increase the inhibitory effects of aspirin on growth in *PIK3CA*-mutated cells. (**a**) Schematic of the sequential enzymatic reactions of glutamine metabolism with inhibitors for each step. (**b**,**c**) Combined growth inhibition of *PIK3CA*-mutated cells treated with aspirin (ASA) and GLS inhibitor. HCT-15 cells (**b**) were treated with 2 mM ASA and/or 10 µM CB-839 for 96 hr. HCT116 cells (**c**) were treated with 2 mM ASA and/or 10 nM CB-839 for 72 hr. (**d**,**e**) Combined growth inhibition of *PIK3CA*-mutated cells treated with ASA and the GOT/GPT inhibitor. HCT-15 (**d**) and HCT116 (**e**) cells were treated with 2 mM ASA and/or 5 mM aminooxyacetate (AOA) for 72 hr. (**f**,**g**) Combined growth inhibition of *PIK3CA*-mutated cells treated with ASA and xCT inhibitor. HCT-15 cells (**f**) were treated with 2 mM ASA and/or 1 mM salazosulfapyridine (SASP) for 72 hr. HCT116 cells (**g**) were treated with 2 mM ASA and/or 0.5 mM SASP for 72 hr. The data obtained with dimethyl sulfoxide (DMSO) were taken as 100%. Cell viability was measured using the Cell Counting Kit-8 assay. The data obtained with DMSO-treated controls were taken as 100%. Columns, means (*n* = 3); bars, s.d. ** *p* < 0.01, significantly different from the DMSO-treated control. Statistical analyses were performed using a two-tailed *t*-test (**b–g**).

As recently reported, altered glutaminolysis may be a therapeutically targetable vulnerability of cancer [24]. During glutaminolysis, glutaminase (GLS) and aminotransferases (GOT and GPT) convert glutamine to α-ketoglutarate (αKG), which eventually enters the TCA cycle to supply biosynthetic processes. In addition, glutamate regulates the homeostasis of intracellular reactive oxygen (ROS) with the cystine/glutamate exchange transporter (xCT, coded by the *SLC7A11* gene; Figure 5a). We thus hypothesized that aspirin-induced glutaminolysis should be suppressed in order to further sensitize cells to aspirin. First, we used CB-839, which is an orally bioavailable inhibitor of GLS, to be combined with aspirin. The combination of aspirin with CB-839 resulted in more significant inhibition of cell growth than each single agent in HCT-15 (Figure 5b) and HCT116 (Figure 5c) cells. Next, cotreatment of aspirin with aminooxyacetate (AOA), a compound that inhibits the enzymatic activity of GOT and GPT, exhibited combined effects on growth inhibition in both cell lines (Figure 5d,e). Lastly, salazosulfapyridine (SASP), an inhibitor of xCT, also led to less cell growth than each agent alone when combined with aspirin (Figure 5f,g). These results suggest that inhibition of the enzymes or transporter involved in the glutamine pathway increase the efficacy of aspirin in *PIK3CA*-mutated cells.

### 2.6. Cotreatment of Aspirin with Glutaminolysis-Targeting Agents Exhibits Combinatorial Effects in Long-Term Incubation

**Figure 6:**
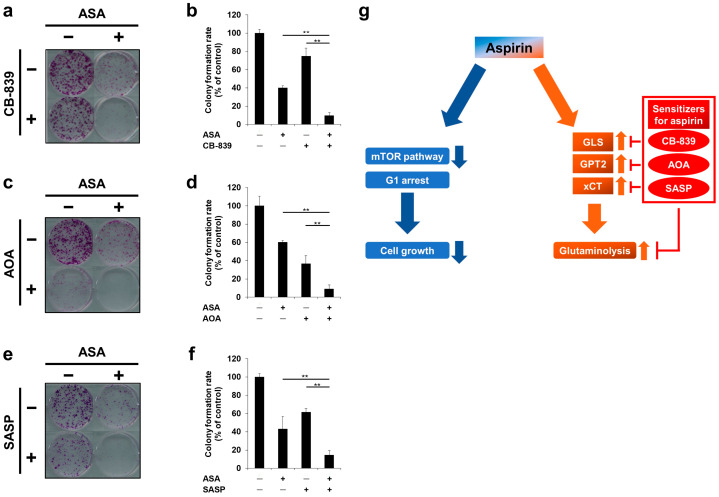
Cotreatment of aspirin with glutaminolysis-targeting agents exhibited combinatorial effects during long-term incubation of *PIK3CA*-mutated cells. (**a**,**b**) The colony formation of *PIK3CA*-mutated cells treated with aspirin (ASA) and GLS inhibitor. HCT-15 cells were treated with 2 mM ASA and/or 10 µM CB-839 for 6 days. (**c**,**d**) The colony formation of *PIK3CA*-mutated cells treated with ASA and GOT/GPT inhibitor. HCT-15 cells were treated with 2 mM ASA and/or 5 mM aminooxyacetate (AOA) for 6 days. (**e**,**f**) The colony formation of *PIK3CA*-mutated cells treated with ASA and xCT inhibitor. HCT-15 cells were treated with 2 mM ASA and/or 0.5 mM salazosulfapyridine (SASP) for 6 days. The representative images of stained colonies are shown (**a**,**c**,**e**). Colony numbers were calculated by comparing with the dimethyl sulfoxide (DMSO)-treated control (**b**,**d**,**f**). Columns, means (*n* = 3); bars, s.d. Statistical analyses were performed using a two-tailed *t*-test (**b**,**d**,**f**). (**g**) Schematic representation of two different aspects of the antitumor activity of ASA in *PIK3CA*-mutated cells. In addition to inhibiting the mTOR pathway and inducing G1 arrest, ASA increases glutaminolysis and sensitizes *PIK3CA*-mutated cells by targeting glutaminolysis.

We also examined the combinatorial effects of aspirin with each glutaminolysis-targeting agent during long-term incubation of cells. Similar to the results of cell viability assays during a drug exposure period of 72 or 96 hr (Figure 5d–g), the combination of aspirin with CB-839, AOA or SASP resulted in more significant inhibition of colony formation than a single agent alone in HCT-15 cells (Figure 6a–f). Based on these data, aspirin-mediated promotion of glutaminolysis via the upregulation of GLS, GPT2 and xCT may make *PIK3CA*-mutated cells vulnerable to glutaminolysis-targeting agents.

## 3. Discussion

In the present study, we demonstrated that aspirin had two different aspects of antitumor activity in *PIK3CA*-mutated CRC cells: (i) inhibitory effects on the mTOR pathway and the induction of G1 arrest and (ii) promotion of glutaminolysis and combinatorial effects with glutaminolysis-targeting agents (Figure 6g). Although aspirin has been recently focused on for its antitumor effects, especially for CRC patients bearing *PIK3CA* mutations, which are expected to be a predictive marker to stratify responders to aspirin treatment [5], we first clarified aspirin-mediated metabolic perturbation of glutaminolysis, which may be targetable to further sensitize *PIK3CA*-mutated cells to aspirin.

The antitumor mechanisms of action of aspirin have been investigated from the perspective of both cyclooxygenase-2 (COX-2)-dependent and independent manners [25]. As mRNA expression of COX-2 was not detected in HCT-15 [26] or HCT116 cells [27], the aspirin-mediated antitumor activity observed in our study was independent of COX-2 expression.

Our study may improve our understanding of why aspirin administration is associated with increased survival for patients with *PIK3CA*-mutated CRC [5]. Some previous reports led to possible interpretations for this clinical question. A recent report demonstrated that G1 arrest-inducing ability of aspirin is stronger in cells with *PIK3CA* mutations than those with wild type using mathematical modeling with in vitro assays [28]. Another report clearly showed that aspirin more markedly induced not only G1 arrest but also apoptosis in *PIK3CA*-mutated CRC cells than wild type cells [29]. We also found that aspirin induced G1 arrest by inhibiting the mTOR pathway in *PIK3CA*-mutated cells (Figure 2), consistent with previous observations [30,31]. As the mTOR pathway is downstream of PI3K, aspirin-mediated mTOR inhibition may explain why *PIK3CA*-mutated cells are more sensitive to aspirin than wild type *PIK3CA* cells. On the other hand, our novel finding that aspirin promotes glutaminolysis may require a new explanation. Consistent with recent reports [14,32], *PIK3CA*-mutated cells needed glutamine to proliferate (Figure 1a,b). We also found that aspirin-mediated growth inhibitory effects were dependent on the presence of glutamine in *PIK3CA*-mutated cells (Figure 1d–f). We therefore speculated that aspirin treatment might be useful for cancers that require glutamine for progression such as *PIK3CA*-mutated CRC, although preclinical and clinical studies are needed.

We found that aspirin and glutamine deprivation induced the same phenotypes such as G1 arrest (Figure 2a,b), inhibition of the mTOR pathway (Figure 2d,e) and upregulation of ATF4 (Figure 4c) in *PIK3CA*-mutated CRC cells. Consistently, RNA-seq analysis showed that gene sets related to glutamine depletion were enriched in aspirin-treated *PIK3CA*-mutated cells (Figure 3b and Figure S4a,b). These data imply that aspirin may deplete glutamine in the medium, whose mechanism may be explained by the expression of the *SLC1A5* gene (coding ASCT2, neutral amino acid transporter-2) after aspirin treatment (Figure 3c,d). That is, considering that glutamine is transported into the cell by ASCT2, aspirin-induced ASCT2 may promote glutamine uptake, resulting in depletion of glutamine in the medium, although further studies including metabolites tracing analysis are needed to address the precise mechanisms involved in this process.

Another novel finding taken from our study is that aspirin upregulated ATF4 expression at the mRNA level (Figure 4b), which may partially account for the increased glutaminolysis. Although it is well known that ER stress promotes the translation of ATF4 mRNA, we did not observe the phosphorylation of eIF2α (Figure 4d), which is a representative marker of ER stress. A recent report demonstrated that mTORC1 controls ATF4 expression by regulating both the translation and stability of its mRNA [33]; however, aspirin inhibited the mTOR pathway in our study (Figure 2d,e). Of note, glutamine deprivation led to the upregulation of ATF4 mRNA under activation of the KRAS-PI3K axis [22]. Considering that aspirin phenocopied the effects of glutamine deprivation (Figure 2, Figure 3b, Figure 4c and Figure S4a,b) in *PIK3CA*-mutated HCT-15 cells, which also has *KRAS* mutation, aspirin may perturb glutamine metabolism with upregulating ATF4 mRNA analogous to glutamine deprivation, whose mechanism(s) should be further investigated.

Aspirin-mediated promotion of glutaminolysis may provide novel therapeutic insights. In the present study, we examined three candidate agents with aspirin: GLS, GOT/GPT and xCT inhibitors. CB-839 has been reported to be effective for several types of solid cancers [34,35] and hematological malignancies [36,37] and many clinical trials of CB-839, including targeting *PIK3CA*-mutated CRC (ClinicalTrials.gov identifier: NCT02861300), are ongoing. Moreover, GLS was reported to increase following mTOR inhibition, thereby providing rationale for the combination of a GLS inhibitor with an mTOR inhibitor [38,39]. Therefore, aspirin-mediated mTOR inhibition resulted in the upregulation of GLS independently of ATF4 (Figure 3c,d) and exhibited combined effects on cell growth with GLS inhibition (Figure 5b,c and Figure 6a,b). As for GOT/GPT inhibitors, GPT2 inhibition is expected to be an effective approach to treat *PIK3CA*-mutated CRC [15]. Salazosulfapyridine (SASP), which is in the phase I study as a specific xCT inhibitor, increased the progression-free survival of patients with advanced non-small cell lung cancer by targeting CD44v-positive cancer stem cells [40]. Of note, a recent report demonstrated that combined inhibition of xCT and the mTOR pathway resulted in synergistic effects on cell growth [41], further supporting that SASP enhanced the efficacy of “mTOR inhibiting” aspirin. Thus, aspirin may increase the metabolic vulnerability to glutaminolysis inhibition, providing a rationale for combining aspirin with glutaminolysis-targeting agents. As our study was limited to *PIK3CA*-mutated CRC cell lines growing in culture, in vivo experiments using mice xenograft of *PIK3CA*-mutated CRC cells including PDX models are needed to lead to translation of these combinations to the clinic.

## 4. Materials and Methods

### 4.1. Cell Lines and Culture

Human colon cancer cell lines HCT-15, HCT116 and SW480 were obtained from the American Type Culture Collection. SW48 *PIK3CA* (E545K/+) and SW48 *PIK3CA* WT were obtained from Horizon Discovery (Waterbeach, UK). The authenticity of each cell line was confirmed by short tandem repeat profiling at each cell bank. All cell lines were confirmed to be negative for mycoplasma infection using the MycoAlert Mycoplasma Detection Kit (Lonza, Rockland, ME, USA) and used within three months after thawing. All cells were cultured in RPMI-1640 supplemented with 10% fetal bovine serum, 2 mM glutamine, 50 U/mL of penicillin and 100 μg/mL of streptomycin. The culture medium was used within two weeks to avoid degradation of L-glutamine. Cells were incubated at 37 °C in a humidified atmosphere of 5% CO_2_.

### 4.2. Reagents

Aspirin, aminooxyacetate (AOA) and salazosulfapyridine (SASP) were obtained from Sigma (Saint Louis, MO, USA). CB-839 was obtained from Cayman Chemical (Ann Arbor, MI, USA). These reagents were dissolved in the solvent dimethyl sulfoxide (DMSO) as stock solutions and diluted to each working concentration in culture medium. Each agent was prepared at the stock concentration of 1 M (aspirin), 200 mM (SASP) and 10 mM (CB-839).

### 4.3. Cell Viability Assay

Cell viability was measured by a Cell Counting Kit-8 assay (Dojindo, Kumamoto, Japan) according to the manufacturer’s instructions. Briefly, cells were seeded in 96-well plates (2.5–5.0 × 10^3^ cells/well in 100 μL) and cultured overnight. The next day, cells were treated with 100 μL of each diluted drug in a total volume of 200 μL. After incubating the cells for indicated times, the kit reagent WST-8 was added to the medium and incubated for 4 hr. The absorbance at 450 nm of the samples was measured using SpectraMax iD5 (Molecular Devices, LLC, San Jose, CA, USA). All experiments shown were replicated at least twice.

### 4.4. Cell Cycle Analysis and the Detection of Cell Death

Cells were seeded in 6-well plates (5 × 10^4^ cells/well in 2 mL). After overnight culture, each medium was removed, and cells were treated with 2 ml of each diluted drug. After incubating the cells for indicated times, cells were harvested by trypsinization. After centrifugation at 20,400× *g* at 4 °C for 5 min, cells were suspended in PBS containing 0.1% Triton X-100 and 25 μg/mL of propidium iodide. Stained cells were analyzed using FACSCalibur (Becton Dickinson, Franklin Lakes, NJ, USA). Data were analyzed using the Modfit LT software (Becton Dickinson) and Cell Quest software (Becton Dickinson). DNA fragmentation was quantified by the percentage of hypodiploid DNA as the sub-G1 population. All experiments shown were replicated at least twice.

### 4.5. Protein Isolation and Western Blotting

Cells were lysed with lysis buffer containing 50 mM Tris-HCl, 1% sodium dodecyl sulfate, 1 mM dithiothreitol and 0.43 mM 4-(2-Aminoethyl) benzenesulfonyl fluoride hydrochloride. The lysates were sonicated and centrifuged at 20,400× *g* at 4 °C for 20 min, and the supernatant was collected. Equal amounts of the protein extract were subjected to sodium dodecyl sulfate-polyacrylamide gel electrophoresis (SDS-PAGE) and transferred to a polyvinylidene difluoride membrane (EMD Millipore, Billerica, MA, USA). The following were used as the primary antibodies: rabbit anti-human S6K (#2708), phospho-S6K (Thr389) (#9234), anti-human 4E-BP1 (#9644), phospho-4E-BP1 (Ser65) (#9451), ATF4 (#11815), ASCT2 (#8057), phospho-eIF2α (Ser51) (#9721), eIF2α (#9722) antibodies (Cell Signaling Technology, Inc., Danvers, MA, USA), mouse anti-human β-actin antibody (A5441; Sigma), mouse anti-human ASNS antibody (G-10; Santa Cruz Biotechnology, Santa Cruz, CA), rabbit anti-human GPT2 (16757-1-AP) antibody (Proteintech Group, Inc., Chicago, IL, USA), rabbit anti-human Glutaminase (ab156876) and xCT(ab37185) antibodies (Abcam, Cambridge, UK). Signals were detected with Chemi-Lumi One L (Nacalai Tesque, Kyoto, Japan) or Immobilon Western Chemiluminescent HRP Substrate (EMD Millipore). All experiments shown were replicated at least twice.

### 4.6. RNA Isolation and Real-Time Quantitative Reverse Transcription-PCR

Total RNA was isolated from cells treated with each drug using Sepasol-RNA I (Nacalai Tesque) according to the manufacturer’s instructions. Total RNA was reverse transcribed to complementary DNA (cDNA) in a 20 μL reaction volume using the High Capacity cDNA Reverse Transcription Kit (Applied Biosystems, Foster City, CA, USA). An equivalent volume of cDNA solution was used for quantitative RT–PCR. cDNA was amplified using a QuantStudio 3 Real-Time PCR System (Thermo Fisher Scientific, Waltham, MA, USA) with TaqMan Probes for ATF4 (Hs00909569_m1), ASNS (Hs04186194_m1), GLS (Hs00248163_m1), GPT2 (Hs00370287_m1), SLC7A5 (Hs00185826_m1), SLC7A11 (Hs00921938_m1) and β2MG (Hs00984230_m1; Applied Biosystems). The expression of each mRNA was normalized to that of β2MG mRNA in the same sample. All experiments shown were replicated twice.

### 4.7. Microarray Analysis

Microarray datasets of DLD-1 cells treated with dimethyl sulfoxide (DMSO) or 3 mM aspirin for 24 hr were obtained from the supplemental information of a previous report [20]. The top 1000 upregulated genes were applied to enrichment analysis. Metascape (http://metascape.org/gp/index.html#/main/step1; accessed on 20 February 2020) was used for enrichment analysis [42]. We designated the following genes as glutaminolysis-related genes: *GLS*, *GLS2*, *GLUL*, *GLUD1*, *GLUD2*, *ASNS*, *GOT1*, *GOT2*, *GPT1*, *GPT2*, *SLC1A5*, *SLC7A5* and *SLC7A11*.

### 4.8. RNA Sequencing

Total RNA was extracted from cells using RNeasy Mini Kit (Qiagen) and processed using TruSeq Stranded mRNA Sample Prep Kit (Illumina, San Diego, CA, USA). Poly(A) RNA libraries were then constructed using TruSeq Stranded mRNA Library Preparation Kit (Illumina, San Diego, CA, USA), and sequenced at 100 bp paired-ends on an Illumina NovaSeq6000 platform. Sequencing data in FASTQ format will be provided during review.

### 4.9. RNA-Seq Data Analysis

RNA-seq reads were then quantified using ikra v1.2.2 [43], an RNA-seq pipeline centered on Salmon [44]. Ikra automates the RNA-seq data analysis process, which includes quality control of reads (Trim Galore version 0.4.1 [45] with Cutadapt version 1.9.1 [46]) and transcript quantification (Salmon version 0.14.0 with reference transcript sets in GENCODE release 31 for human). These tools were used with default parameters in ikra. Count tables were imported into integrated Differential Expression and Pathway analysis (iDEP v0.90), an integrated web application for pathway analysis of RNA-Seq data [47]. The quantified transcript reads were filtered with 0.5 counts per million (CPM) in at least one sample and transformed, using EdgeR: log2(CPM + c), with pseudocount c = 4. Gene set enrichment analysis (GSEA) was performed as part of the iDEP software with fold-change values returned by DESeq2. False positive rate (FDR) *q*-value < 0.2 were considered as enriched and were further investigated.

### 4.10. Motif Enrichment Analysis in Promoter Sequences of the Glutaminolysis-Related Genes

Genomic sequences 1000 bases upstream of the annotated transcription starts of RefSeq genes (human genome assembly GRCh38/hg38) were obtained from the UCSC genome browser (http://hgdownload.soe.ucsc.edu/goldenPath/hg38/bigZips/). Promoter sequences for the glutaminolysis-related genes were extracted from the promoter sequences and used for analysis of transcription factor-binding motif enrichment. AME software (http://meme-suite.org/) [48] and HOCOMOCO v11 [49], a dataset of transcription factor-binding motifs obtained from a large ChIP-seq dataset, were used to perform the analysis and calculate the *p*-value for motif enrichment.

### 4.11. Colony Formation Assay

Cells were seeded at a density of 2000 cells per well in 6-well plates. After incubating for 24 hr, the cells were treated with each agent. The cells were fixed with 10% formalin and stained with 0.1% crystal violet after incubation for another 6 days. The area of stained colonies was quantified using ImageJ from the National Institutes of Health (Bethesda, MD, USA).

### 4.12. Statistical Analysis

All data are presented as the mean ± s.d. The significance of differences in the means among three or more groups was analyzed by one-way analysis of variance using Tukey post-hoc tests, and that of comparisons between two groups was tested using the two-tailed unpaired Student’s *t*-test. A value of *p* < 0.05 was considered to be significantly different from each control.

## 5. Conclusions

We demonstrated aspirin-mediated inhibition of the mTOR pathway and the promotion of glutaminolysis, further supporting the relationship between aspirin efficacy and mutated status of the *PIK3CA* gene. This study also provides a plausible approach to increase the antitumor efficacy of aspirin by targeting glutaminolysis in *PIK3CA*-mutated CRC.

## Supplementary Materials

The following are available online at https://www.mdpi.com/2072-6694/12/5/1097/s1, Figure S1: Glutamine dependency of the inhibitory effect of aspirin on cell growth in *PIK3CA* wild type and -mutated cells, Figure S2: The sub-G1 population after aspirin treatment with or without glutamine, Figure S3: Heatmaps using gene sets of “GO biological process” derived from the RNA-seq analysis of *PIK3CA*-mutated cells treated with aspirin, Figure S4: Heatmaps using gene sets of “Molecular Signatures Database (MSigDB) Curated Gene Sets” derived from the RNA-seq analysis of *PIK3CA*-mutated cells treated with aspirin, Figure S5: Differential enrichment analysis derived from the microarray analyses of aspirin-treated cells, Figure S6: The uncropped Western blot bands used in this study.
