# Supplementary material for: Deactivation of Glutaminolysis Sensitizes PIK3CA-Mutated Colorectal Cancer Cells to Aspirin-Induced Growth Inhibition
Source: Cancers (Basel). 2020 Apr 30;12(5):1097. doi: 10.3390/cancers12051097 (PMC7281071; doi:10.3390/cancers12051097)
Supplement: Supplementary file 1 [file cancers-12-01097-s001.pdf]

Supplementary Materials: Deactivation of Glutaminolysis Sensitizes *PIK3CA*-Mutated Colorectal Cancer Cells to Aspirin-Induced Growth Inhibition

Shogen Boku, Motoki Watanabe, Mamiko Sukeno, Takeshi Yao, Kiichi Hirota, Mahiro Iizuka-Ohashi, Kyoko Itoh and Toshiyuki Sakai

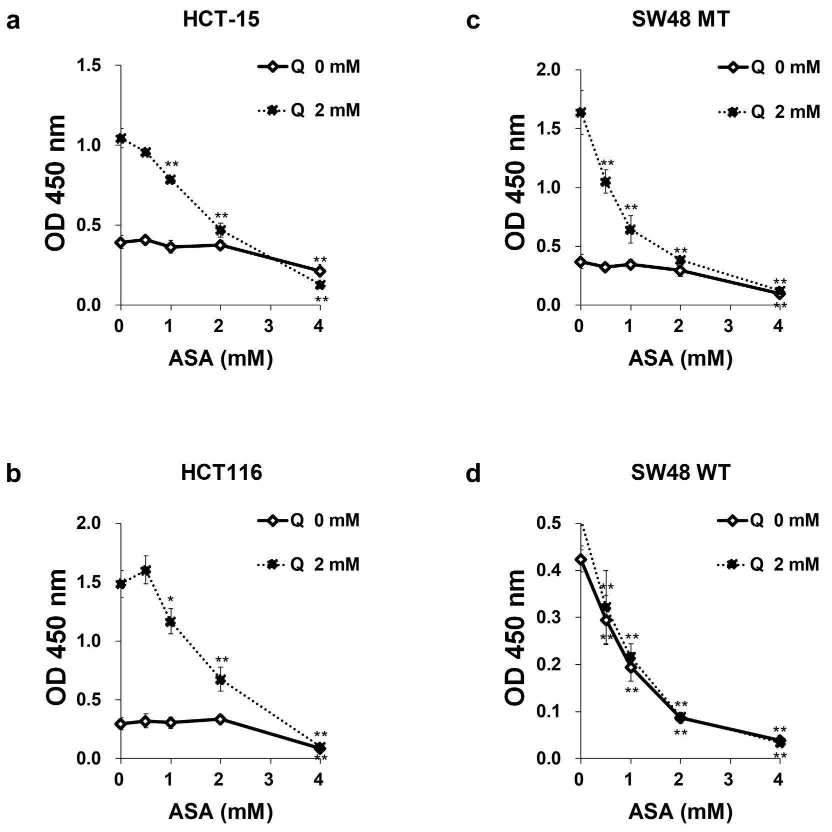

Figure S1. Glutamine dependency of the inhibitory effect of aspirin on cell growth in *PIK3CA* wild type and -mutated cells. The optical density at 450 nm is displayed on the Y-axis. HCT-15 (**a**), HCT116 (**b**), SW48 MT (*PIK3CA* MT) (**c**) and SW48 WT (*PIK3CA* WT) (**d**) cells were treated with aspirin (ASA) at the indicated concentrations for 72 hr with or without 2 mM glutamine (Q). Columns, means (n = 3); bars, s.d. * p <0.05, ** p < 0.01, significantly different from the DMSO-treated control. Statistical analyses were performed using one-way ANOVA with Tukey's post-hoc test (**a**–**d**).

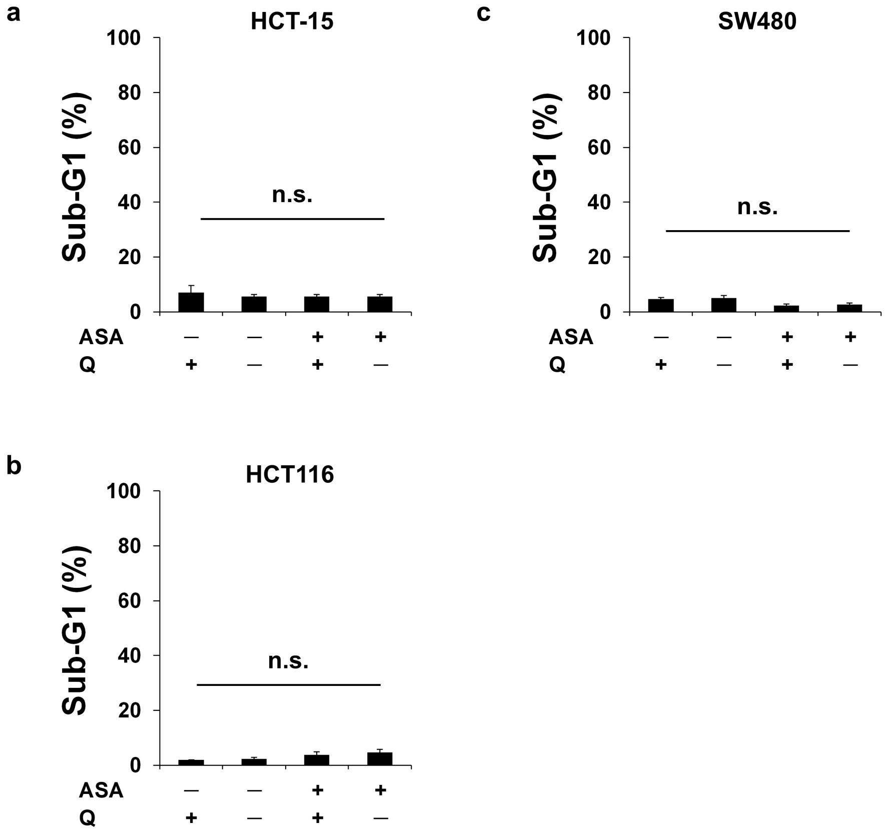

Figure S2. The sub-G1 population after aspirin treatment with or without glutamine. HCT-15 (**a**), HCT116 (**b**) and SW480 (**c**) cells were treated with DMSO or 2 mM aspirin (ASA) with or without glutamine (Q) for 48 hr. DNA contents of the cells were analyzed by flow cytometer. Columns, means of triplicate data; bars, SD. Statistical analyses were performed using two-tailed *t*-test (**a**–**c**).

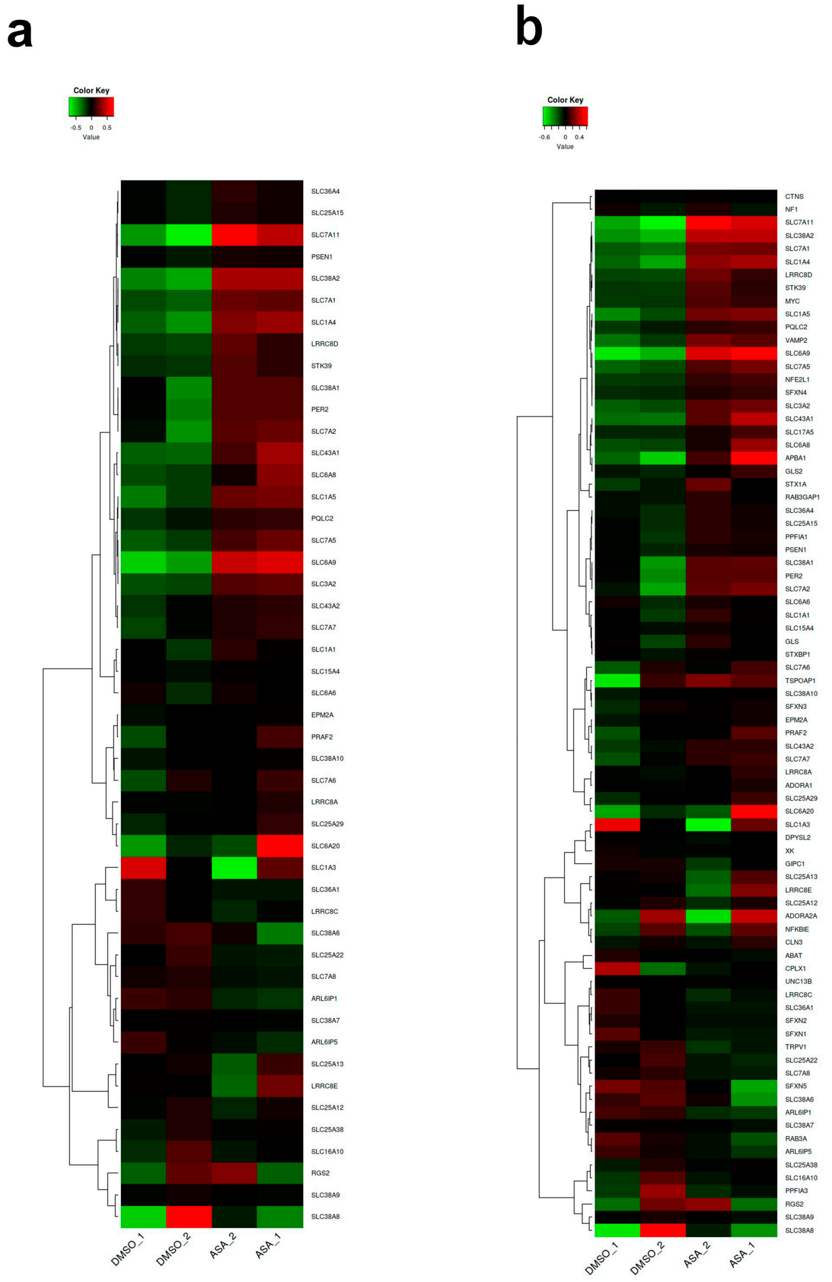

Figure S3. Heatmaps generated from iDEP using gene sets of "GO biological process" derived from the RNA-seq analysis of *PIK3CA*-mutated cells treated with aspirin. "Amino acid transmembrane transport" (**a**) and "Amino acid transport" (**b**) were shown. HCT-15 cells were treated with dimethyl sulfoxide (DMSO) or 2 mM aspirin (ASA) for 24 hr.

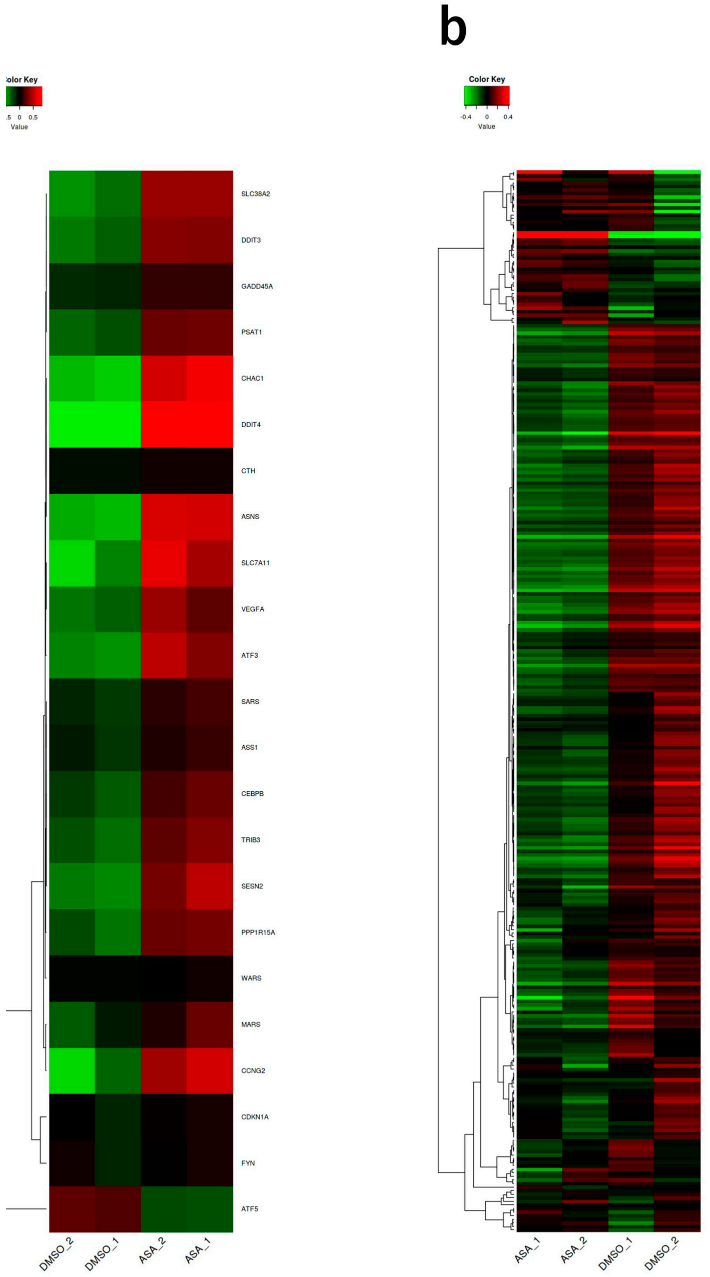

Figure S4. Heatmaps using gene sets of "Molecular Signatures Database (MSigDB) Curated Gene Sets" derived from the RNA-seq analysis of *PIK3CA*-mutated cells treated with aspirin. "KRIGE_AMINO_ACID_DEPRIVATION" (**a**) and "PENG_GLUTAMINE_DEPRIVATION_DN" (**b**) were shown. HCT-15 cells were treated with dimethyl sulfoxide (DMSO) or 2 mM aspirin (ASA) for 24 hr.

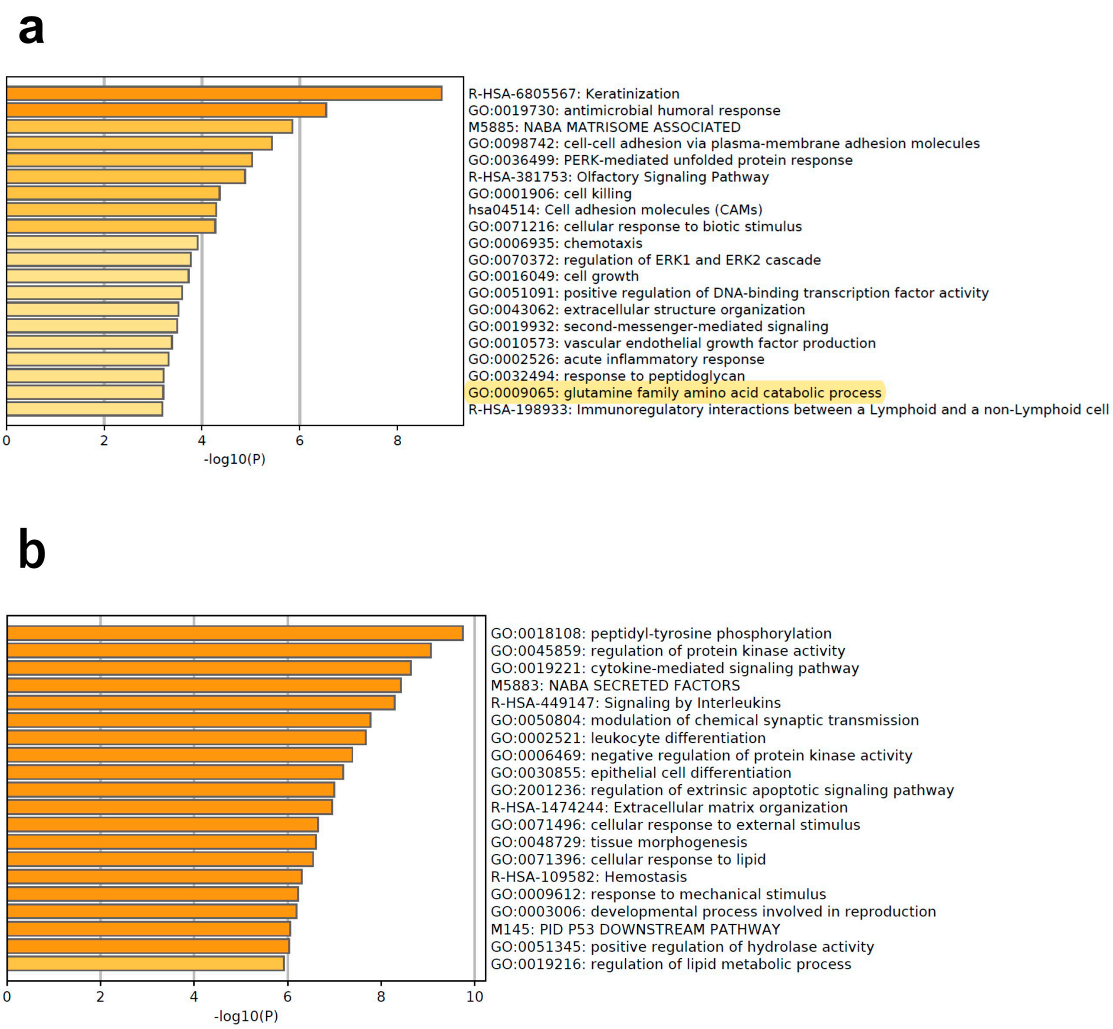

Figure S5. Differential enrichment analysis derived from the microarray analyses of aspirin-treated cells. The top 20 enrichment pathways are displayed on the Y-axis. (**a**) The microarray dataset of DLD-1 cells (*PIK3CA* MT) treated with dimethyl sulfoxide (DMSO) or 3 mM aspirin (ASA) for 24 hr was obtained from the supplemental information of a previous report [20]. (**b**) That of SW620 cells (*PIK3CA* WT) treated with DMSO or 5 mM ASA for 24 hr. The top 1,000 upregulated genes were applied to enrichment analysis and colored to indicate the P-values. Metascape was used for enrichment analysis.

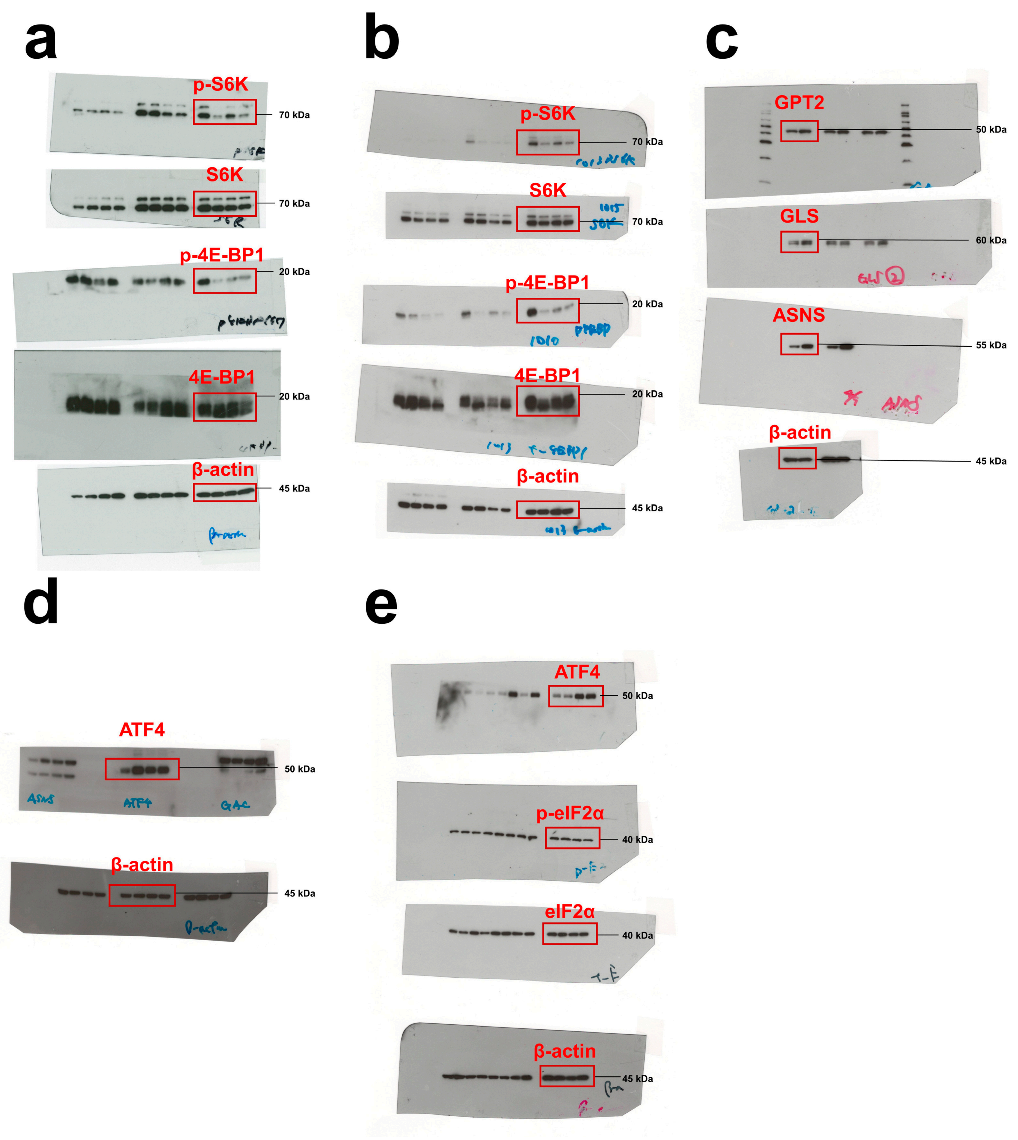

Figure S6. The uncropped Western blot bands used in this study. (**a**) The uncropped Western blot bands related to Figure 2d. (**b**) The uncropped Western blot bands related to Figure 2e. (**c**) The uncropped Western blot bands related to Figure 3e. (**d**) The uncropped Western blot bands related to Figure 4c. (**e**) The uncropped Western blot bands related to Figure 4d.

© 2020 by the authors. Licensee MDPI, Basel, Switzerland. This article is an open access article distributed under the terms and conditions of the Creative Commons Attribution (CC BY) license (http://creativecommons.org/licenses/by/4.0/).
